# Mental health needs of defendants with intellectual disabilities presenting at court

**DOI:** 10.1192/j.eurpsy.2024.68

**Published:** 2024-08-27

**Authors:** J. McCarthy, E. Chaplin, D Harvey, K Marshall-Tate, S Ali, A Forrester

**Affiliations:** ^1^King’s College London, London, United Kingdom; ^2^University of Auckland, Auckland, New Zealand; ^3^ London South Bank University, London, United Kingdom

## Abstract

**Background:**

Studies in different countries of defendants with mild to borderline intellectual disability found they have distinct characteristics from other defendants. The aim of this study was to examine several characteristics among defendants with intellectual disability comparing to those defendants without intellectual disability presenting to court services in London, England.

**Method:**

This was a retrospective data analysis of routine administrative data collected by the Liaison and Diversion services across five Magistrates courts in London, England. Data were analysed on defendants identified through screening to have an intellectual disability and compared to defendants without an intellectual disability.

**Results:**

9088 defendants were identified and of these 349 (4%) had an intellectual disability. Defendants with intellectual disability were over four times more likely to have comorbid attention deficit hyperactive disorder and over 14 times more likely to have autism spectrum disorder. There was an increased odds ratio of self-reported suicidal/self-harming behaviour for those defendants with intellectual disability compared to those without intellectual disability.

**Conclusion:**

This study has highlighted the increased vulnerability of defendants with intellectual disability for other neurodevelopmental disorders.

**Disclosure of Interest:**

J. McCarthy Grant / Research support from: Guy’s & St. Thomas’ Charity for £674,000, E. Chaplin Grant / Research support from: Guy’s & St. Thomas’ Charity for £674,000

